# Radiological Diagnosis of Congenital Diaphragmatic Hernia in 17th Century Korean Mummy

**DOI:** 10.1371/journal.pone.0099779

**Published:** 2014-07-02

**Authors:** Yi-Suk Kim, In Sun Lee, Go-Un Jung, Myeung Ju Kim, Chang Seok Oh, Dong Su Yoo, Won-Joon Lee, Eunju Lee, Soon Chul Cha, Dong Hoon Shin

**Affiliations:** 1 Department of Anatomy, Ewha Womans University School of Medicine, Seoul, Korea; 2 Department of Radiology, Seoul National University Hospital, Bundang, Korea; 3 Department of Anatomy, Dankook University College of Medicine, Chonan, Korea; 4 Department of Anatomy, Seoul National University College of Medicine, Seoul, Korea; 5 Institute of Forensic Science, Seoul National University College of Medicine, Seoul, Korea; 6 Department of Diagnostic Radiology, Dankook University College of Medicine, Chonan, Korea; 7 Department of Internal Medicine, Asan Medical Center, Seoul, Korea; 8 Dongguk Institute of Cultural Properties, Daegu, Korea; Ohio State University, United States of America

## Abstract

Congenital diaphragmatic hernia (CDH) is a birth defect of the diaphragm resulting in pulmonary sequelae that threaten the lives of infants. In computed tomography (CT) images of a 17th century middle-aged male mummy (the *Andong* mummy), we observed that the abdominal contents had protruded into the right thoracic cavity through the diaphragmatic defect, accompanied by a mediastinal shift to the left. On autopsy, the defect in the right posterolateral aspect of the diaphragm was reconfirmed, as was the herniation of the abdominal organs. The herniated contents included the right lobe of the liver, the pyloric part of the stomach, a part of the greater omentum, and the right colic flexure connecting the superior part of the ascending colon and the right part of the transverse colon. Taking our CT and autopsy results together, this case was diagnosed as the Bochdalek-type CDH. Herein we make the first ever report of a CT-assisted diagnosis of a pre-modern historical case of CDH. Our results show the promising utility of this modality in investigations of mummified human remains archaeologically obtained.

## Introduction

A diaphragmatic hernia is a partial protrusion of abdominal organs through a defect in the diaphragm. Congenital diaphragmatic hernia (CDH) is caused by a failure in the fusion of the pleuroperitoneal folds that manifests as a defect of the diaphragm during fetal development [1]–[4]. The Bochdalek-type CDH is a protrusion through the posterolateral part of the diaphragm [5]–[8]. Considering that the left side of the pleuroperitoneal canal closes later than does the right side, it is understandable that CDH is much more frequently found on the left side of the diaphragm [4], [7], [9].

Bochdalek CDH occurrence differs by generation. It is known to occur in 1/3600–1/7000 infants or neonates, and potentially develops into life-threatening cardiopulmonary complications [6], [10]–[13]. In adults by contrast, due to its asymptomatic status, Bochdalek CDH was very difficult to diagnose until the advent of effective radiological diagnostics [14]–[18].

Reports of CDH in anatomical investigations of archaeologically obtained remains are rare. In fact, since CDH can be diagnosed only by observation of an abnormal arrangement of abdominal contents in the thoracic cavity [7], [8], [19], most cases of skeletonized remains are inapplicable to such studies. In this regard, mummified remains can be very useful [3]. For instance, researchers identified an esophageal hernia of the stomach in a Chilean colonial Indian mummy dating to 1550 CE [3]. Munizaga et al. (1978) concluded that an Atacamena Indian mummy dating to the 3^rd^ century CE might have died of complications from jejunum strangulation caused by herniation into the thoracic cavity. This remains the sole Bochdalek CDH case ever reported on the basis of an archaeological investigation [4].

Over the past several years, 16^th^ to 19^th^ century Korean mummies have come to be recognized as excellent subjects for studies on pre-modern Korean peoples and societies. In well-preserved Korean mummies, we have discovered various pathologies that have elucidated the health and disease status of peoples living during the Joseon period (1392–1910 CE) [20]–[36].

Most recently, in the course of a routine computed tomography (CT) and associated autopsy on a 17th century Korean mummy, we found evidence suggesting that the man might have suffered from CDH. To our knowledge at least, this is the first-ever report of an historical CDH case diagnosed by the combined use of CT and autopsy.

## Materials and Methods

### The mummy

**Figure 1:**
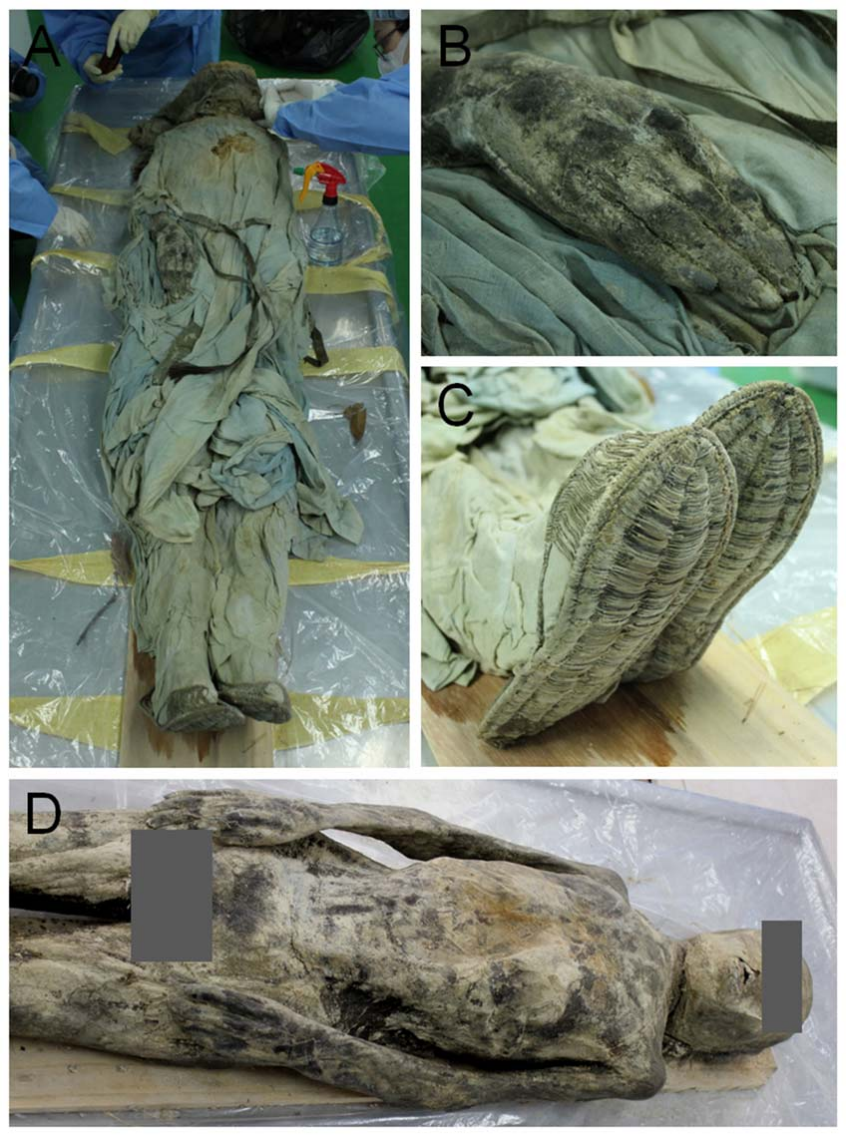
Examination of Andong mummy. (A) Removal of clothing. (B) and (C) Very well preserved human and cultural remains. (B) is hand; (C) is straw shoes. (D) The mummy examined in this study.

On January 2013, a mummy (nick-named the *Andong* mummy) was discovered in the Joseon tomb at Andong, a southeastern Korean city. In the coffin, the mummy was lying on the back with the face up. By carbon dating, this case was estimated to be 230±30 BP (conventional radiocarbon age: 1783±30 CE). Under the auspices of the Dongguk Institute of Cultural Properties, the mummy was moved to our lab for further study (**Figure 1**). Our anatomical, histological and radiological investigations were authorized by the Institutional Review Board (IRB), Seoul National University Hospital (H-1108-049-120).

Prior to the commencement of our gross examination, textile specialists removed the mummy's shrouds. We then performed anthropometric measurements on the mummy, following the *Standards for Data Collection from Human Skeletal Remains* [37]. Age was estimated by Lamendin's method [38] using a single-root tooth (the right maxilla canine).

### Computed tomography (CT)

The whole body CT scanning was performed before *post-factum* autopsy. The tool used here is 64MDCT scanner (VCT, GE healthcare). CT scans were obtained with a 0.625 mm thickness and 0.625 mm interval from head to toe. Images were reviewed with lung, bone and soft tissue settings on multi-planar format (axial, sagittal, and coronal images) on a work station (Advantage Windows Workstation 4.3, GE healthcare). In total, 2465 axial scans were utilized in this study.

For the purposes of comparisons with the current CDH case, we employed CT images of another Joseon-era Korean mummy. He was found in a Joseon tomb on November of 2007 (nick-named the *Gangneung* mummy). According to historical documentation, a male was born in 1561 and died in 1622 at the age of 61. The CT-image-scanning conditions prevailing in this case were already reported in one of our previous studies [30]. Our previous investigations of this case confirmed the lack of any evidence of CDH [23], [39].

#### Post-factum autopsy

For *post-factum* confirmation of our CT image findings, we performed an autopsy on the *Andong* mummy. An initial bell-shaped incision had been made along the outside borders of the anterior torso (**Data S1**). We then carefully examined the diaphragm for any abnormalities and the presence of herniated visceral organs.

## Results

### Anthropological Data

The mummy appeared to be quite well-preserved overall (**Figure 1**). A sex examination, on the basis of evidence of external genitalia, determined the individual to be a male. A topknot clearly noticeable on the head of the mummy (not permitted for bachelors during the Joseon period) established the man's married status. The man was estimated to have died at around 45.4 years of age (**Data S2**). His stature was 160.2 cm by direct anthropometric measurement. The relevant anthropometric data are summarized in **Data S3**. On gross examination on the skin in this case, we could find no pathological changes.

### CT Image Analysis

**Figure 2:**
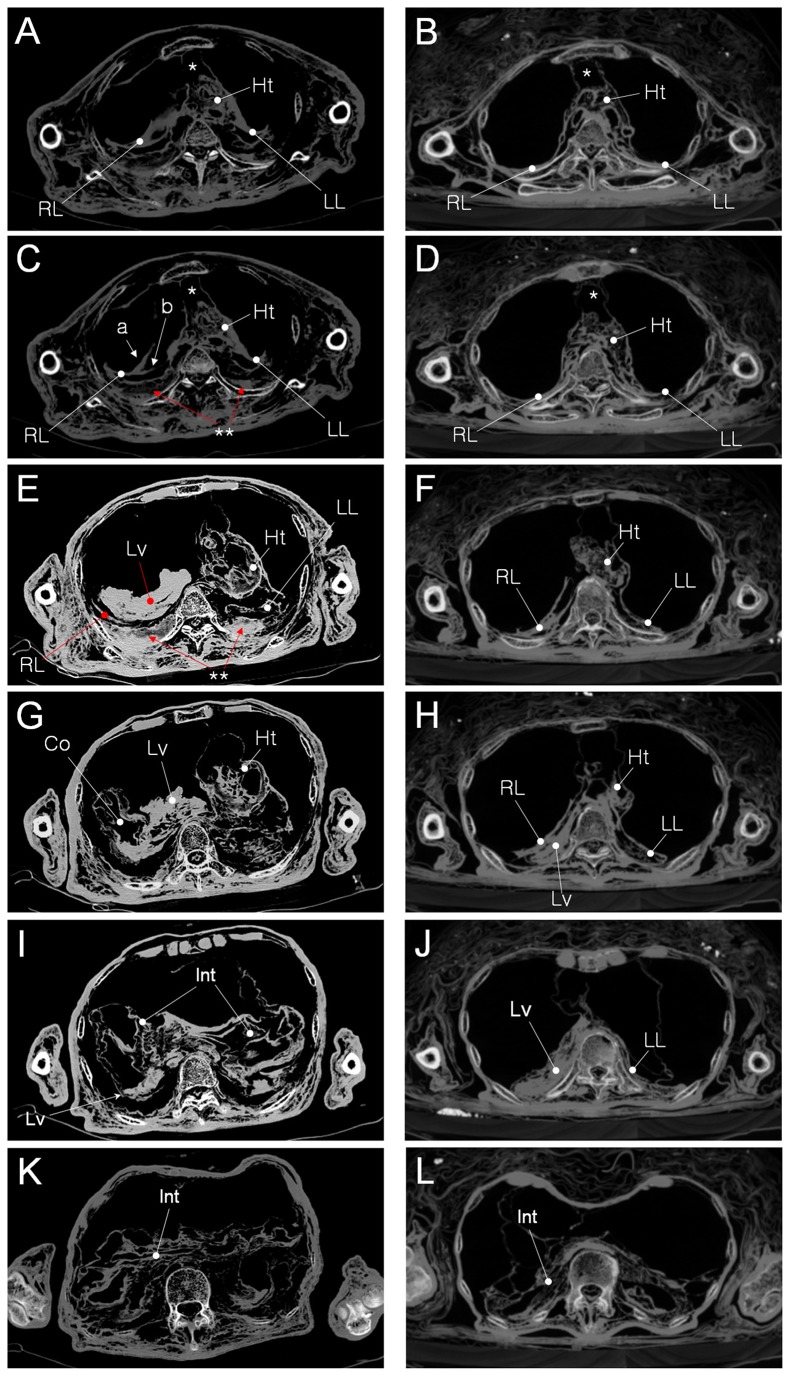
Axial CT images of mummies. Left column (A, C, E, G, I and K): the current Andong case with Congenital Diaphragmatic Hernia (CDH); Right column (B, D, F, H, J and L): Gangneung mummy case without CDH. (A) and (B) TV3 upper level. Ht, heart; RL, right lung; LL, left lung; asterisk, pericardial sac. (C) and (D) TV3 lower level. RL in (C) showed split pattern (a and b). Ht in (C) exhibited mediastinal shift to the left. (D) The similar structure is not seen at the same CT level of Gangneung mummy. (E) and (F) TV5 level. (E) Liver (Lv) is seen at the same level of heart (Ht). Mediastinal shift to the left side is very severe, comparing with normal CT in (F). (G) and (H) TV7 level. (G) Colon (Co) and Liver (Lv) is seen at the same level of heart (Ht). Mediastinal shift is still remarkable. (H) Colon is not observed. Liver (LV) begins to emerge. (I) and (J) TV 9 level. (I) Mainly intestine (Int) is visible. (J) Large liver (LV) could be seen at the same level of Gangneung mummy. (K) and (L) LV1 level. Intestine or other mummified abdominal organs fills the cavity in both CT images.

As on CT images of other Korean mummies [23], [29], [30], [33], [35], the cranial, thoracic and abdominal organs in the *Andong* case were sunken to the dorsal side, probably as a results of the long-term effect of gravitational force. On the axial CT images, the location and morphology of the *Andong* mummy's internal organs were generally similar to those of the *Gangneung* mummy (**Figure 2**). However, the *Andong* mummy showed unique anomalous thoracic-cavity structures not seen in the *Gangneung* mummy: specifically, the position of the presumptive liver, at the level of the heart, was much too high (**Figure 2**).

**Figure 3:**
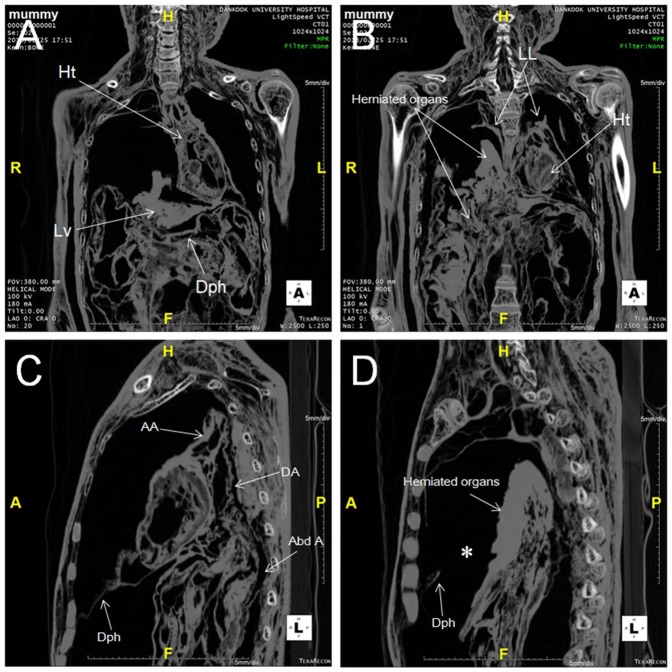
Coronal CT images of the current Andong mummy with Congenital Diaphragmatic Hernia. (A) Herniated liver (Lv) could be seen while diaphragm (Dph) is visible underneath. (B) Herniated organs stretching from abdominal to thoracic cavities could be clearly seen. Ht, heart; LL, lung. (C) and (D) Sagittal CT images of Andong mummy. (C) Diaphragm (Dph) is clearly seen between thoracic and abdominal cavity. (D) Herniated organs could be observed through the diaphragmatic defect (asterisk). AA, ascending aorta; DA, descending aorta; Abd A, abdominal aorta.

The reformatted sagittal and coronal images also showed herniated organs. There was a large diaphragmatic defect, through which organs were herniated into the right thoracic cavity (**Figure 3**). We speculated that the herniated organs were the liver and bowel.

### Autopsy

**Figure 4:**
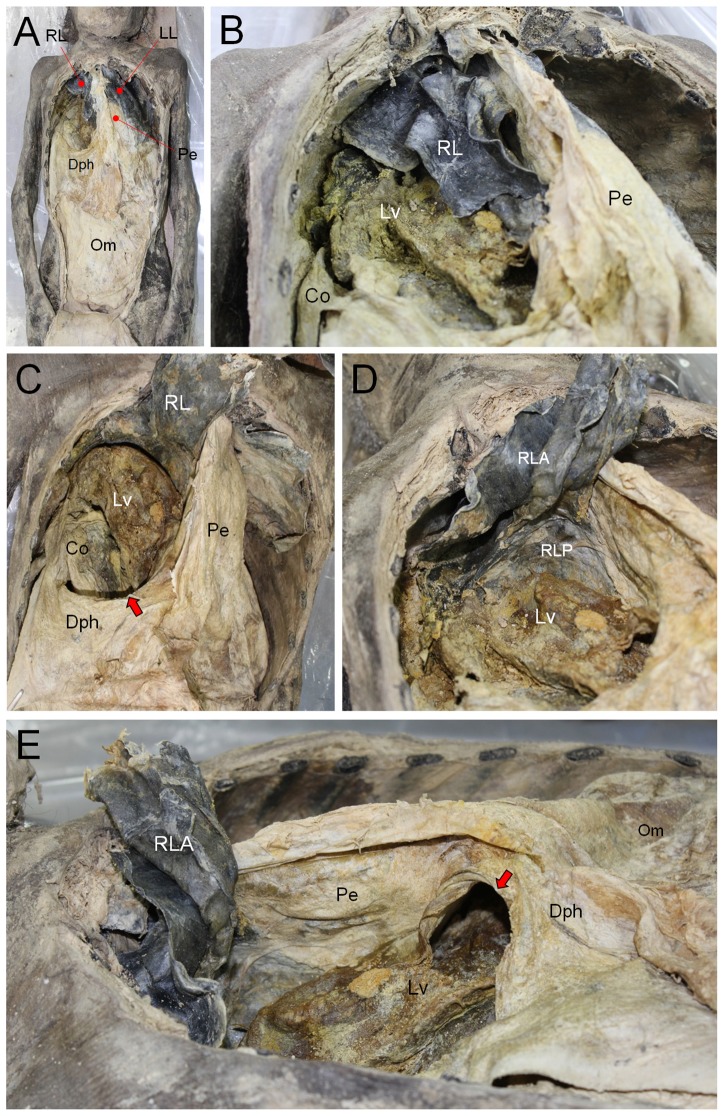
Dissection of the mummy. (A) Thoracic and abdominal cavities exposed. Pe, pericardium; Dph, diaphragm; Om, omentum; Pe, pericardium; LL, Left lung; RL, right lung. (B) Magnified image of right thoracic cavity. Parts of liver (Lv) and colon (Co) could be found within the right thoracic cavity. (C) A part of RL is turned back. Lv and Co are much clearly identified. The defect in diaphragm is indicated by arrow. (D) Lv is protruding into the diaphragmatic surface of RL that is therefore folded into anterior (RLA) and posterior parts (RLP). (E) View from cephalic to rostral. Bochdalek hernia (indicated by arrow) in Dph is observed. Lv protrudes through the hernia defect.

Our autopsy of the *Andong* mummy revealed no specific injury pattern such as bone fracture. Because the defect (12 cm×8 cm) was positioned in the right posterolateral aspect of the diaphragm, the Morgagni- and central-type hernias could be excluded. We also found a contracted lung in the right thoracic cavity, possibly caused by persistent upward pressure from the protrusion of the herniated abdominal organs (**Figure 4A, 4B**). In this circumstance, the herniated organs were trapped in the lung indentation (**Figure 4C, 4E**). The herniated contents in the right thoracic cavity were recognized as the right lobe of the liver, the pyloric part of stomach, a part of the greater omentum, and the right colic flexure constituting the superior part of the ascending colon and the right part of the transverse colon (**Figure 4D, 4E** **&** **Table 1**).

**Table 1:** Herniated organs of the mummy in this study.

Sample	Herniated Organs		Specific Part of Organs
Gagok-ri	Solid organ	Liver	Right lobe
	Hollow organ	Stomach	Pyloric part
		Large intestine	Greater omentum
			Transverse colon
			Ascending colon

**Figure 5:**
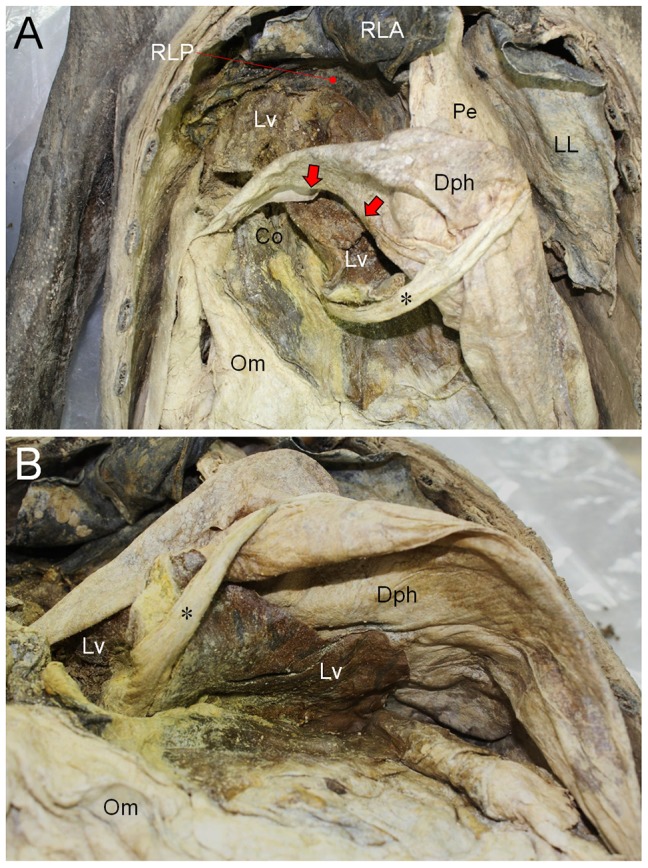
Dissection view from abdomen of the mummy. (A) Diaphragmatic hernia (indicated by arrows) could be seen in the right side of diaphragm (Dph). Liver (Lv) and Colon (Co) are running thorough the diaphragmatic defect. RLA, anterior part of indented right lung; RLP, posterior part of indented right lung; LL, left lung; Om, omentum; Pe, pericardium. Ligamentum teres hepatis is marked by asterisk. (B) Left part of diaphragm (Dph). View from below. Liver (Lv) is also observed in the subdiaphragmatic area. No defects identified in diaphragm.

By contrast, with respect to the left lung or the left part of the diaphragm of the *Andong* mummy, we could find no pathologic abnormalities (**Data S4**). Other abdominal contents, including the spleen, small intestine, round ligament, pancreas, left lobe of the liver, and others, were all in the correct positions, showing no evidence of herniation (**Figure 5A, 5B**). No trauma-, strangulation-related or any other complications were discovered in the thoracic or abdominal cavity of the *Andong* mummy.

The heart of the mummy, however, was found in a displaced position. We searched for any coronary abnormalities, particularly as congenital changes (e.g. right-to-left shunt through foramen ovale or ductus arteriosus) or acquired ones (e.g. right ventricular-wall hypertrophy due to pulmonary hypoplasia) are known to be caused by Bochdalek hernia [40]–[42]. There were no suspicious signs of pathology in the atria or ventricles.

## Discussion

Nowadays, non-invasive CT is recommended as the primary diagnostic tool for archaeologically obtained, mummified human remains worldwide [33], [43]. The rapid advancements in the technique have made possible the obtainment of high-quality images of mummies, thus enabling precision non-invasive identification of any pathological changes. As non-invasive study by techniques such as CT becomes more commonly used and recommended for diagnosis of mummy pathologies, valuable data and experience have been accumulated from historical mummy cases.

The diagnostic utility of CT imaging notwithstanding, confirmation of the diseases in mummy cases remains difficult. The reason is that mummified organs, due to the long-term effects of dehydration and gravitational force, have distorted morphologies and are atypically located. CT images of mummified organs therefore show quite different patterns from those in the cases of living patients [33]. In this regard, the accumulation of CT data is essential to improved diagnostic accuracy in different cases of mummy diseases.

Unfortunately, however, CDH reports from anatomical studies on mummies are still very rare, the only exceptions being a few New World mummy cases. Moreover, in all of the previously reported CDH cases, the sole diagnostic tool was a simple autopsy. Therefore, CT would not enable differentiation of mummy CDH cases in which abdominal organs had in fact herniated through the diaphragmatic defect.

In this regard, the present study is meaningful to concerned researchers. Actually, a certain diagnosis of Bochdalek CDH could be made in the case of a 17th century middle-aged mummy (the Andong mummy) uniquely subjected to combined CT/autopsy examination. The CT images and autopsy results showed right-sided CDH, which type is less prevalent than left-sided instances [19], [44]. In fact, this is the first ever report of a CT-assisted diagnosis of a pre-modern historical case of CDH.

In our current investigation, we noted that the liver can be a uniquely useful organ for diagnosis of right-sided CDH in mummies, as its S-shaped (curved) pattern and uniform radio-density on CT images are maintained, even in cases of herniation into the thoracic cavity (**Data S5**). By using the mummified liver as a kind of landmark, we speculated that the herniated contents on our CT images had stretched through the diaphragmatic defect and protruded further into the thoracic cavity.

In some previously reported cases of modern living CDH patients, CT images showed that the ipsilateral lung became hypoplastic by displacement of the abdominal organs into the thoracic cavity, while the mediastinum was deviated to the contralateral side [45], [46]. These CDH signs were clearly seen in the Andong mummy of the present study. Actually, the right lung was compressed by the herniation; and the heart was shifted more to the left side of the thoracic cavity. As in other adult CDH cases already reported, it was speculated that this individual might have experienced some difficulties such as shortness of breath, abdominal pain, chest pain, nausea, or vomiting during his lifetime [9], [47].

Most cases of Bochdalek CDH occur in infants or neonates with life-threatening complications, adult cases being relative rarities and asymptomatic [6], [10]–[13], [48]. Therefore, it may seem unusual that our middle-aged subject had passed the critical point for survival, with such a serious Bochdalek CDH. Still, we also admit that thanks to the rapid development of the CT technique, Bochdalek CDH has been detected with increasing frequency in adult patients nowadays [9], [16], [19], [47], [48].

Some adult cases clearly show how well the clinical signs of CDH can be masked as individuals grow into adulthood. One such example is a 50-year-old female patient who complained of mild dyspnea. In this patient, clinicians found very few signs and symptoms suggestive of adult CDH. By CT diagnostics, however, a remarkable displacement of abdominal organs into the thoracic cavity could be detected. Additionally, serious atelectasis was also found in her left lung, while most of the left thoracic cavity was filled by displaced abdominal organs. The herniation was so serious that the patient's heart was shifted to the right, further compressing the right lung. The size of the defect on the diaphragm was as large as 10×8 cm [48]. In fact, this case clearly shows that adult CDH can be very serious even if the patient complains only of mild clinical symptoms.

Actually, Korean mummy of this study, as in the case of that 50-year-old female patient, exhibited a serious degree of CDH along with a mediastinal shift. However, when we searched the intestines for evidence of complications such as perforation or strangulation of herniated organs (the causes of most fatal emergences of adult CDH [15]), we found none. This means that the CDH itself might not have been the main cause of death in his case. He could have lived with CDH in this lifetime while experiencing a few signs of respiratory disturbances. We suspected that the functional defects caused by the CDH in the present male mummy case might have been largely compensated for as he grew older.

## Conclusions

In this study, a Bockdalek-type CDH in a 17th century *Andong* mummy was successfully diagnosed by CT imaging combined with autopsy. In our CT interpretation, the mummified liver, with its unique shape and radio-density, was used as a kind of indicator for the herniation of abdominal organs into the right side of the thoracic cavity. Longer experience with CT imaging diagnostics and the resultant data accumulation will no doubt prove instrumental to the analysis of future mummy CDH cases.

## Supporting Information

Data S1 (A) Bell-shape incision was on mummy. Anterior body wall was turned over back. Pleura and peritoneum are exposed. (B) Dissection of left lung. No herniated organs were found in left thoracic cavity. RL, right lung; LL, left lung; Pe, pericardium.
(TIF)

Data S2 Age estimation based on Lamendin's method (1992).
(DOC)

Data S3 Anthropometric data.
(DOC)

Data S4 (A) Left-sided lung detached from pericardium (Pe). Left lung (LL) becomes very thin. RLA, anterior part of indented right lung. (B) Fully detached left lung.
(TIF)

Data S5 CT images of four different Korean mummies (the current CDH case, Gangneung, Hwasung, and Yangju mummies). Mummified livers (Lv) of each mummy show uniquely curved shapes. Livers of Gangneung, Hwasung and Yangju mummies are present in abdomen. Note the liver of the current CDH case located in thorax.
(JPG)
