# Supplementary figures and images for: Radiological Diagnosis of Congenital Diaphragmatic Hernia in 17th Century Korean Mummy
Source: PLoS One. 2014 Jul 2;9(7):e99779. doi: 10.1371/journal.pone.0099779 (PMC4079512; doi:10.1371/journal.pone.0099779)

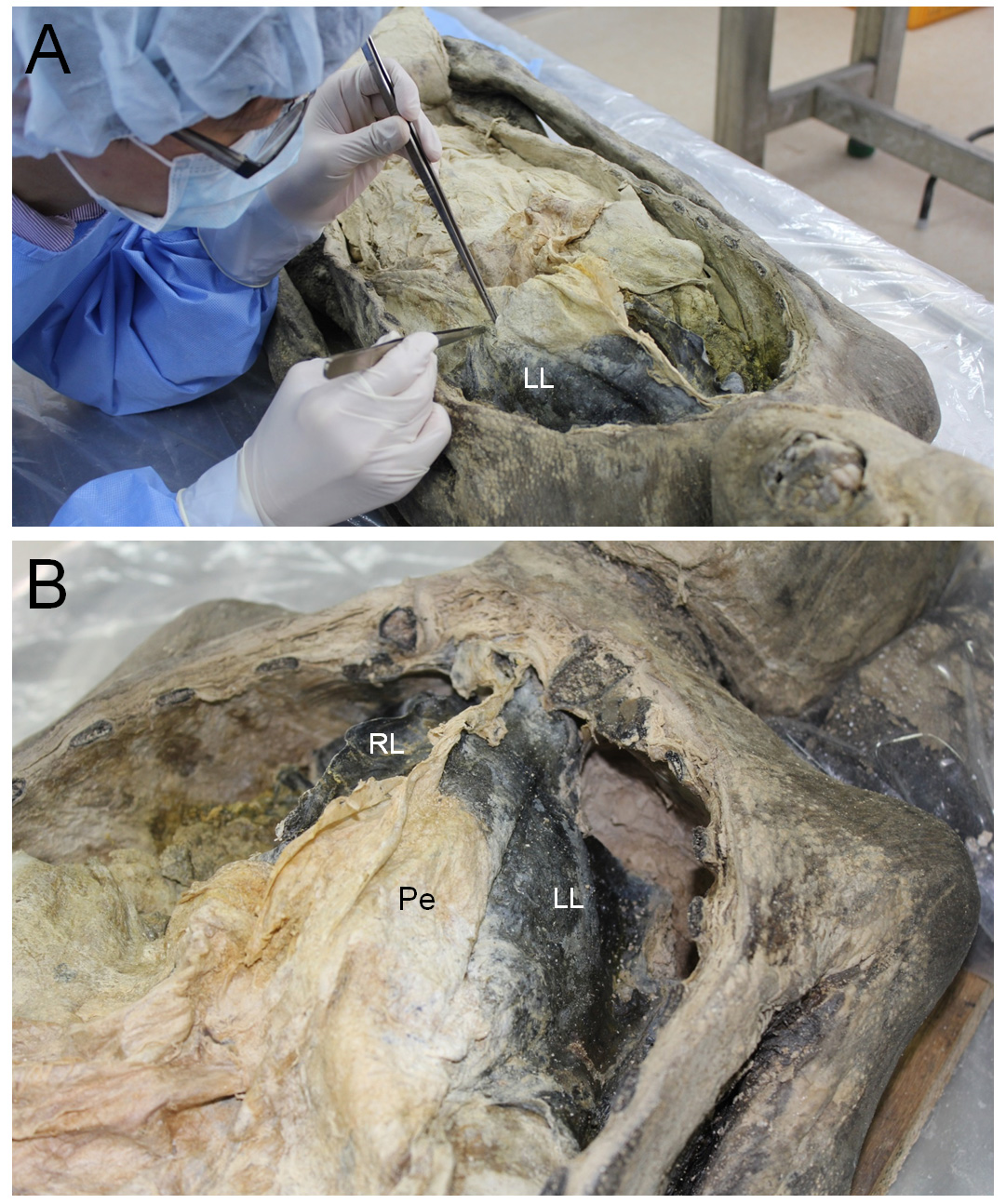

Supplement: Data S1 — (A) Bell-shape incision was on mummy. Anterior body wall was turned over back. Pleura and peritoneum are exposed. (B) Dissection of left lung. No herniated organs were found in left thoracic cavity. RL, right lung; LL, left lung; Pe, pericardium. (TIF) [file pone.0099779.s001.tif]

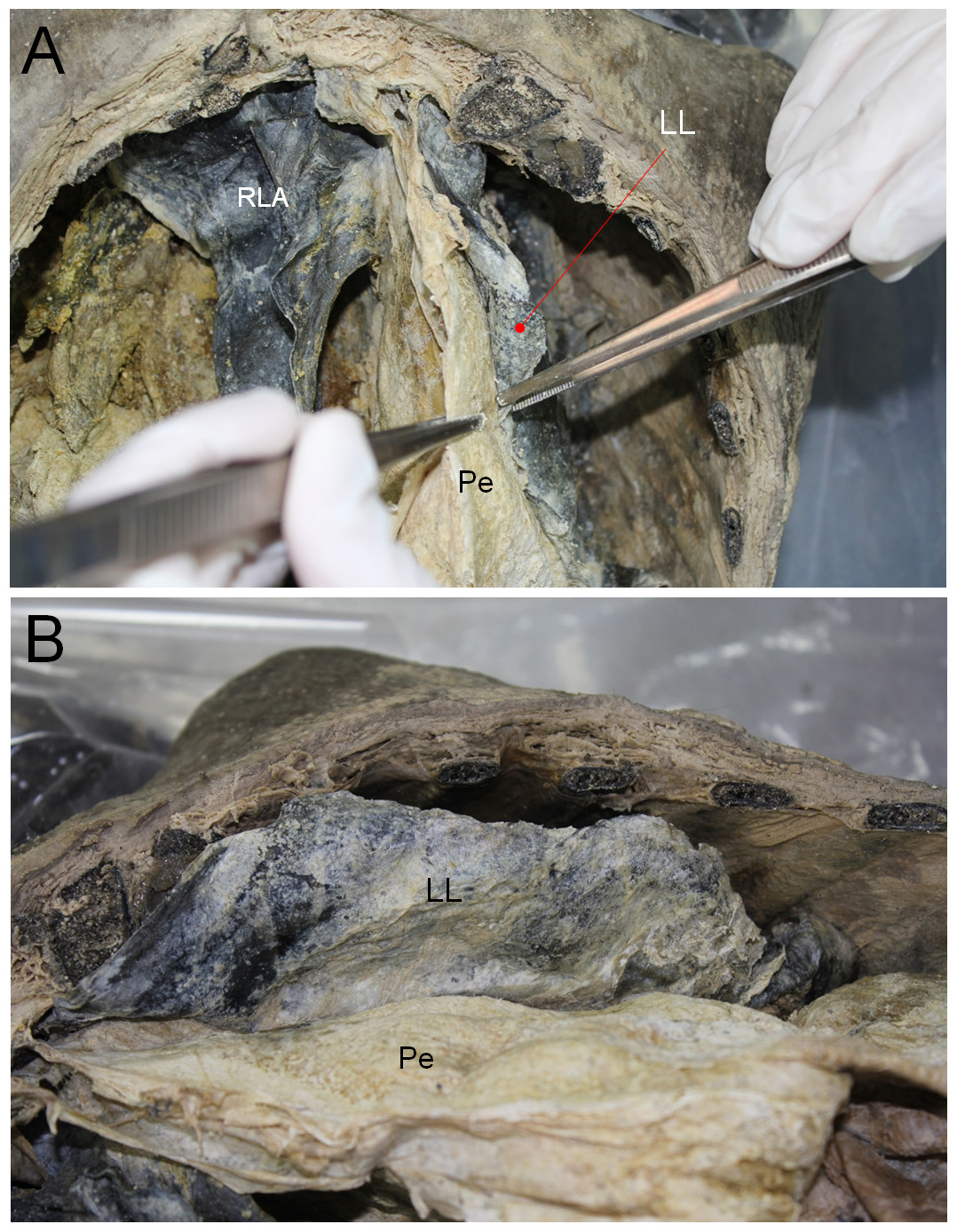

Supplement: Data S4 — (A) Left-sided lung detached from pericardium (Pe). Left lung (LL) becomes very thin. RLA, anterior part of indented right lung. (B) Fully detached left lung. (TIF) [file pone.0099779.s004.tif]

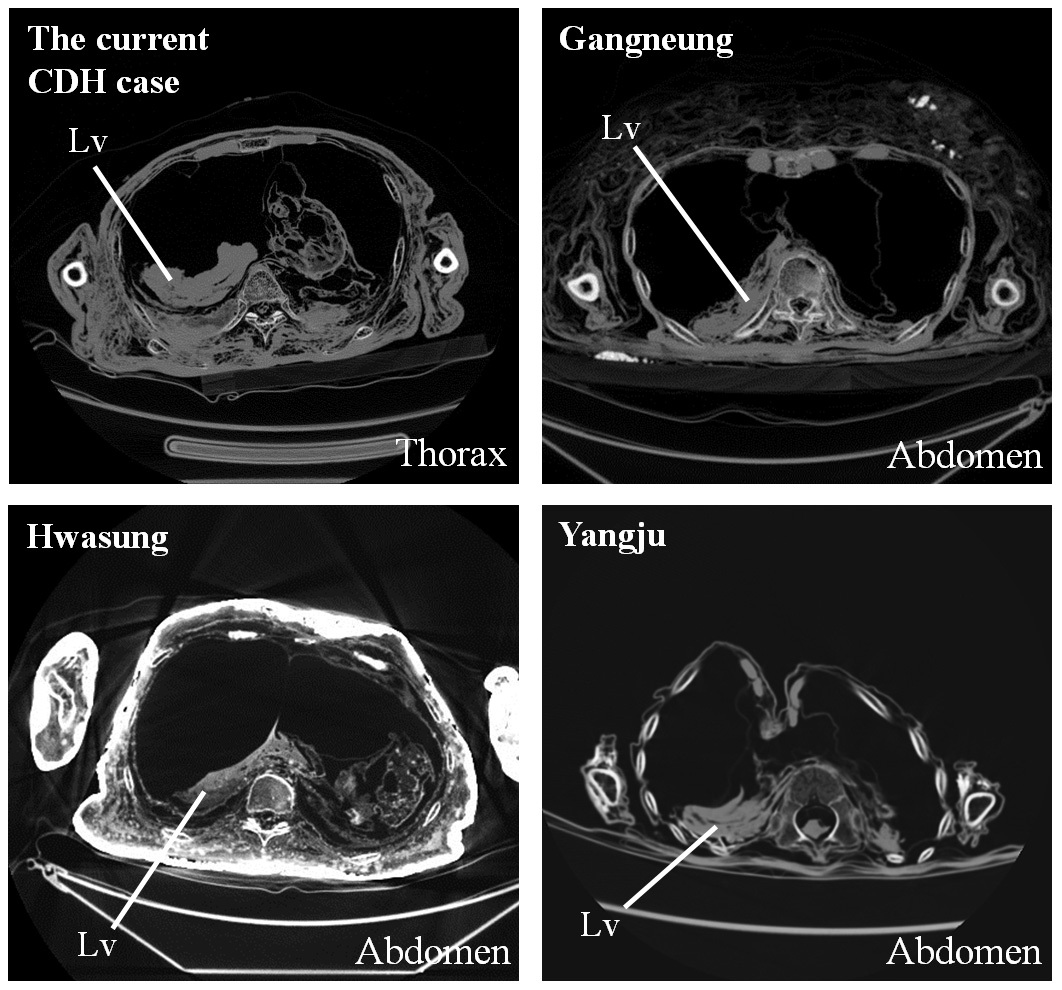

Supplement: Data S5 — CT images of four different Korean mummies (the current CDH case, Gangneung, Hwasung, and Yangju mummies). Mummified livers (Lv) of each mummy show uniquely curved shapes. Livers of Gangneung, Hwasung and Yangju mummies are present in abdomen. Note the liver of the current CDH case located in thorax. (JPG) [file pone.0099779.s005.jpg]
