# Supplementary material for: Radiological Diagnosis of Congenital Diaphragmatic Hernia in 17th Century Korean Mummy
Source: PLoS One. 2014 Jul 2;9(7):e99779. doi: 10.1371/journal.pone.0099779 (PMC4079512; doi:10.1371/journal.pone.0099779)
Supplement: Data S2 — Age estimation based on Lamendin's method (1992). (DOC) [file pone.0099779.s002.doc]

Supplementary Data 2. Age estimation based on Lamendin’s method (1992)

| Sample | Root height | Periodontosis | Trans. height | Estimated Age |
| --- | --- | --- | --- | --- |
| Right maxilla canine | 19.38mm | 3.18mm | 7.79mm | 45.4 yr |
