# Supplementary material for: Radiological Diagnosis of Congenital Diaphragmatic Hernia in 17th Century Korean Mummy
Source: PLoS One. 2014 Jul 2;9(7):e99779. doi: 10.1371/journal.pone.0099779 (PMC4079512; doi:10.1371/journal.pone.0099779)
Supplement: Data S3 — Anthropometric data. (DOC) [file pone.0099779.s003.doc]

Supplementary Data 3. Anthropometric Data (cm)

| item | right | left |
| --- | --- | --- |
| Height | 160.2 |  |
| Anterior trunk height | 126.6 |  |
| Thoracic Height | 14.9 |  |
| Abdominal Height | 36.9 |  |
| Acrominale Height (R/L) | 130.4 | 131.6 |
| Radiale Height (R/L) | 99.6 | 102.0 |
| Stylion Height (R/L) | 80.1 | 81.4 |
| Dactylion Height (R/L) | 65.0 | 67.2 |
| Ilioscristale Height (R/L) | 92.1 | 95.1 |
| Iliospinale Height (R/L) | 82.4 | 84.0 |
| Pubic Symphyseal Height | 75.2 |  |
| Trochanterion Height (R/L) | 79.5 | 81.4 |
| Tibiale Height (R/L) | 37.5 | 39.4 |
| Sphyrion Height (R/L) | 4.2 | 6.1 |
| Biacromial Breadth | 33.8 |  |
| Bicristal Breadth | 35.0 |  |
| Upper Arm Length (R/L) | 31.5 | 31.9 |
| Lower Arm Length (R/L) | 23.2 | 20.9 |
| Total Arm Length (R/L) | 54.7 | 52.8 |
| Total Upper Extremity Length (R/L) | 70.7 | 69.6 |
| Hand Length (R/L) | 16.0 | 16.8 |
| Hand Breadth (R/L) | 8.5 | 8.0 |
| Thigh Height (R/L) | 37.7 | 35.6 |
| Leg Height (R/L) | 33.2 | 33.7 |
| Foot Length (R/L) | 19.9 | 20.0 |
| Foot Breadth (R/L) | 7.4 | 6.8 |
| Width of the Head | 16.0 |  |
| Bitragion Diameter | 15.7 |  |
| Forehead Height | 13.1 |  |
| Height of the Head and Face | 15.9 |  |
| Length of Head | 17.9 |  |
| Circumference of the Head | 56.9 |  |
| Distance Vertex to Tragion (R/L) | 14.1 | 15.2 |
| Width of the Face | 11.8 |  |
| Width of the Mandible | 12.1 |  |
| Height of the face | 12.9 |  |
| Tragion-Glabella Half-Arc (R/L) | 15.4 | 15.3 |
| Tragion-Gnathion Half-Arc (R/L) | 16.4 | 17.2 |
| Anatomical Width of the Nose | 3.7 |  |
